# Genetic, epigenetic and enviromental influencing factors on the regulation of precocious and delayed puberty

**DOI:** 10.3389/fendo.2022.1019468

**Published:** 2022-12-22

**Authors:** Maria Felicia Faienza, Flavia Urbano, Luigi Antonio Moscogiuri, Mariangela Chiarito, Stefania De Santis, Paola Giordano

**Affiliations:** ^1^ Department of Precision and Regenerative Medicine and Ionian Area, University of Bari “Aldo Moro”, Bari, Italy; ^2^ Giovanni XXIII Pediatric Hospital, Bari, Italy; ^3^ Department of Pharmacy-Pharmaceutical Science, University of Bari “Aldo Moro”, Bari, Italy; ^4^ Department of Interdisciplinary Medicine, University of Bari “Aldo Moro”, Bari, Italy

**Keywords:** precocious puberty, delayed puberty, genetic, epigenetic, enviromental factors, endocrine disruptors

## Abstract

The pubertal development onset is controlled by a network of genes that regulate the gonadotropin releasing hormone (GnRH) pulsatile release and the subsequent increase of the circulating levels of pituitary gonadotropins that activate the gonadal function. Although the transition from pre-pubertal condition to puberty occurs physiologically in a delimited age-range, the inception of pubertal development can be anticipated or delayed due to genetic and epigenetic changes or environmental conditions. Most of the genetic and epigenetic alterations concern genes which encode for kisspeptin, GnRH, LH, FSH and their receptor, which represent crucial factors of the hypothalamic-pituitary-gonadal (HPG) axis. Recent data indicate a central role of the epigenome in the regulation of genes in the hypothalamus and pituitary that could mediate the flexibility of pubertal timing. Identification of epigenetically regulated genes, such as Makorin ring finger 3 (*MKRN3*) and Delta-like 1 homologue (*DLK1*), respectively responsible for the repression and the activation of pubertal development, provides additional evidence of how epigenetic variations affect pubertal timing. This review aims to investigate genetic, epigenetic, and environmental factors responsible for the regulation of precocious and delayed puberty.

## Introduction

**Figure 1:**
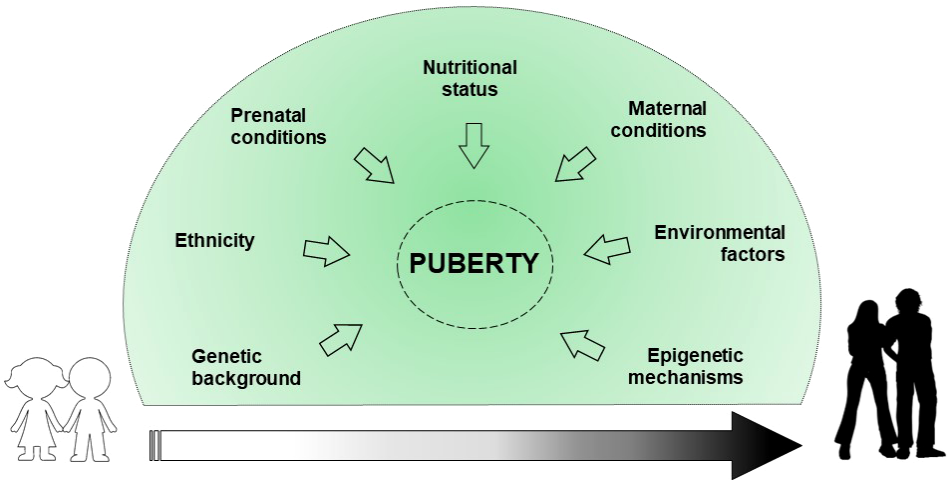
Exogenous and endogenous factors involved in the regulation of the puberty onset.

Puberty represents a significant period in the stages of growth and development that defines the transition from childhood to adulthood due to psycho-physical changes. In addition, the reproductive capacity is acquired. Physiologically, the start of the puberty is caused by the reactivation of signals already developed during fetal life. Indeed, the hypothalamic-pituitary-gonadal axis (HPG) activity ranges from birth to 4-6 months and 2 years, in males and females respectively (1). This phenomenon called “mini-puberty” is due to a decrease in the levels of placental sex hormones, and the resulting loss in negative feedback on gonadotropin releasing hormone (GnRH). After this period, there is a halt of GnRH pulse generator until puberty, which slows reproductive function. The mechanisms that trigger the reinitiating of the GnRH pulse generator and the inception of puberty are not yet clear, although several factors are involved in regulating pubertal timing (2, 3) (**Figure 1**).

Genetic background explains about 50-80% of the variability in pubertal onset and progression (4). Some ethnics groups, particularly African American and Hispanic, show an earlier onset of puberty due to genetic and nutritional factors (5). Prenatal conditions, such as intrauterine growth restriction (IUGR) and small for gestational age (SGA) birth, may affect pubertal development (6). Maternal breastfeeding appears to inhibit the early onset of puberty, mainly due to the positive effect on the childhood overweight (7). Nutritional conditions such as excess of energy intake, macro/micronutrient imbalance and dietary styles can determine the early activation of the HPG axis (7). Childhood obesity may impact on the early onset of pubertal development, albeit no statistical evidence exists on the difference in the age of menarche occurrence between obese and normal weight girls (8). Maternal education, social level, age of menarche occurrence, pre-pregnancy body mass index (BMI), ethnicity, age upon delivery, smoking habits, and alcohol/coffee/tea consumption during pregnancy, are reported to correlate with pubertal timing variations in the offspring (9). Environmental factors, such as substances capable of interfering with the endocrine system (phthalates, dioxins, polybrominated biphenyls, and polychlorinated biphenyls) seem to have a role in influencing pubertal timing (4, 10, 11). Finally, epigenetic mechanisms are assumed to have a central role in regulating the pubertal onset through a balance between repression and activation of gene expression (12).

The aim of this review is to focus on the new insights on genetic, epigenetic, and environmental regulations in the context of precocious and delayed puberty.

## GnRH pulse generator and the KNDy SYSTEM: the role of stimulatory and inhibitory signals

**Figure 2:**
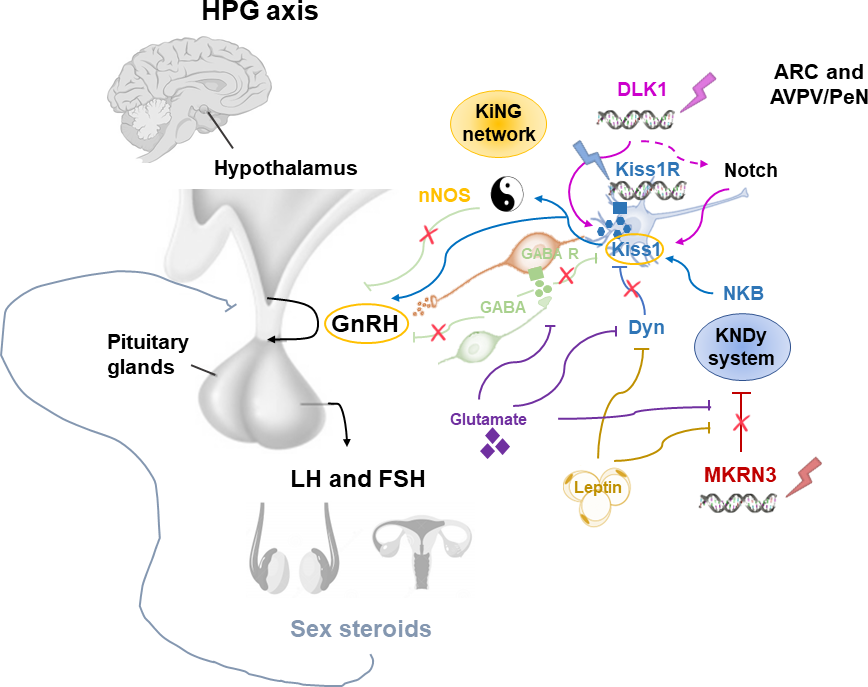
Modulation of the GnRH pulse generator by mechanisms influencing the pubertal timing. ARC, arcuate nucleus; AVPN/PeN, anteroventral periventricular/periventricular nucleus; Dyn, dynorphine A; FSH, Follicle-stimulating hormone; GnRH, Gonadotropin Releasing Hormone; HPG, hypothalamic-pituitary-gonadal; KiNG network, Kisspeptin-nNOS-GnRH; Kiss1, kisspeptin; KNDy, kisspeptin/neurokinin B/dynorphine A; LH, luteinizing hormone; MKRN3, macorin-3; NKB, neurokinin B; nNOS, neuronal nitric oxide synthase.

Pubertal timing is the result of the interaction among hormones, neuronal signals and environmental factors that begins in the earlier stage of development. This interaction leads to the activation of the HPG axis (2, 13). Different hypothalamic factors and excitatory and inhibitory neuronal signals modulate the GnRH pulse generator function (**Figure 2**). KNDy system, which includes kisspeptin/neurokinin B/dynorphine A (KNDy), represents the most important regulator of GnRH secretion. Kisspeptin encoded by the *Kiss1* gene and generated by Kiss1 neurons is the key element of the GnRH pulse generator, together with the neurokinin B and dynorphin A, which exert respectively stimulatory and inhibitory signals that tune kisspeptin oscillation (14). Kiss1 neurons are found in the arcuate nucleus (ARC) and in the anteroventral periventricular/periventricular nucleus (AVPV/PeN) and are controlled by sex gonadal steroids. In the females, AVPV/PeN Kiss1 neurons drive the increase in preovulatory luteinizing hormone (LH) in response to the positive feedback of estradiol. On the other hand, ARC Kiss1 neurons regulate the tonic release of GnRH/LH in response to sex steroid negative feedback, thus sending hormonal, neuroendocrine and metabolic informations (14). Kiss1 neurons has also been recognized in the posterodorsal part of the medial amygdala in mice. These neurons regulate the GnRH pulse generator, as well as influence emotional and sexual behavior, pubertal timing, and ovulation (15, 16). In humans, the role of KNDy system has been clarified by the association of loss-of-function mutations in the kisspeptin (*KISS1*), kisspeptin receptor (*KISS1R*), neurokinin B (*TAC3*), or neurokinin B receptor (*TACR3*) genes and delayed puberty and hypogonadism (17–19). On the other hand, gain of function mutations of *KISS1R* gene have been correlated with precocious puberty (20–22).

Although the KNDy system plays an essential role in the GnRH pulse generator activity, several observations showed that this is not the only system involved in the regulation of pubertal timing (23). Recently, the kisspeptin-nNOS-GnRH or “KiNG” network that is responsible for generating the “GnRH pulse” and “GnRH surge” is emerging among the regulators of pubertal development (24). In fact, nNOS and kisspeptin seem to act as the Yin and Yang, thanks to their ability to integrate and coordinate distinct signals in order to inhibit or promote GnRH secretion, respectively (**Figure 2**). Before the discovery of the crucial role of kisspeptin in the control of GnRH release, *in vitro* and *in vivo* studies identified the nitric oxide (NO) as a key modulator for the GnRH secretion and preovulatory GnRH/LH surge (25, 26). Neurons which express neuronal NO synthase (nNOS) are involved in the modulation of GnRH neuronal excitability and secretion. In mice, nNOS are expressed early in the hypothalamus, suggesting a role of NO in the maturation of GnRH neurons during postnatal life through the regulation of GnRH mRNA expression (27). Knock-out mouse for *NOS1* gene encoding for the nNOS resulted in hypogonadotropic hypogonadism, infertility and dose-dependent defects in olfaction, hearing, and cognition (28). Furthermore, by using a transgenic *Gpr54*-null IRES-LacZ knock-in mouse model, the expression of kisspeptin receptor GPR54 in the nNOS neurons of preoptic region of the hypothalamus has been demonstrated (29). In humans, differently to mice, some kisspeptin neurons of the infundibular nucleus express *NOS1* (30). Recently, *NOS1* loss-of-function mutations have been found in six subjects with congenital hypogonadotropic hypogonadism (CHH), anosmia, hearing loss, and intellectual disability (30). Thus, interactions between kisspeptin and nNOS neurons may play a central role in regulating the hypothalamic–pituitary–gonadal axis *in vivo*.

Among the inhibitory signals that regulate KNDy-GnRH secretion, the Makorin Ring Finger Protein 3 (MKRN3) has a central role, as its expression in the hypothalamic ARC rapidly declines before the onset of puberty, followed by a stable decrease during the pubertal advancement (31, 32).

This is an imprinted gene as the maternal allele is silenced, and only the paternal allele is expressed. Loss-of-function mutations of *MKRN3* gene cause the most cases of familial central precocious puberty (CPP) (33–35). Furthermore, whole genome analysis studies (GWAS) demonstrated that single nucleotide polymorphisms (SNPs) of the MKRN3 region can regulate the age of menarche occurrence in healthy girls (36). Girls with *MKRN3* gene mutations show a more marked advancement in early pubertal signs and at a younger age than boys, indicating a sexually dimorphic effect of MKRN3 on pubertal development (37). Regarding the genotype-phenotype correlation, the median age at diagnosis is lower in patients with more deleterious mutations (stop or frameshift) than those with missense variants (38).

The *MKRN3* gene encodes a protein implicated in ubiquitination and cell signaling. Recently, Li et al. (39) identified the methyl-CpG binding domain (MBD) 3, an epigenetic reader which regulates gene expression, as the target of MKRN3 ubiquitination. MKRN3-mediated ubiquitination attenuates the binding of Poly(A)-binding proteins (PABPs), which regulate the stability of RNA messengers, to the poly(A) tails of mRNA. Therefore, the poly(A) tail-length of Gonadotropin-Releasing Hormone 1 (GNRH1) mRNA is shortened, and the formation of translation initiation complex (TIC) is compromised. Three members of PABPs (PABPC1, PABPC3 and PABPC4) have been identified as novel substrates for MKRN3. Thus, MKRN3 epigenetically regulates the transcription of *GNRH1* gene through conjugating poly-ubiquitin chains on MBD3 (39). The MKRN3 ubiquitination of MBD3 disrupts the interaction between MBD3 and the DNA demethylase ten eleven human translocation methylcytosine dioxygenase, 2 (TET2), as well as the MBD3 binding to *GNRH1* promoter, thus epigenetically silencing the *GNRH1* transcription and inhibiting puberty initiation (39).

Adipokines like leptin and other factors, such as glutamate and glial signaling molecules are also implicated in the control of GnRH secretion. These activator signals are enhanced by the loss of inhibitory signals within the ARC such as gamma aminobutyric acid (GABA), dynorphin A and MKRN3, resulting in positive feedback on GnRH pulse generator (23). GABA is the most important neurotransmitter which inhibits GnRH release during childhood by both indirectly acting on neurons connected to the GnRH neuronal network, or directly stimulating GnRH neurons through activation of GABA receptors alpha1-subunit (40). GABA receptors are expressed on GnRH neurons; thus, GABA antagonists increase GnRH secretion, leading to early menarche (41).

Furthermore, experimental studies have shown that the hypothalamic GABA tone inhibition leads to precocious puberty, and SNPs of the GABA signaling are related with the age at menarche (42).

## Genetic regulation of central precocious puberty

The first CPP-associated gene alteration was an activating mutation (Arg386Pro) in the G protein coupled receptor 54 (GPR54), also referred to as KISS1R, which binds the kisspeptin (20). This mutation prolongs the reactivity to kisspeptin by decreasing the degradation of KISS1R (43). Two heterozygous missense mutations in the ligand, the kisspeptin, encoded by the gene *KISS1*, were identified in unrelated subjects affected with idiopathic CPP. This variant resulted in a higher kisspeptin resistance to degradation compared with the wild type, determining greater kisspeptin bioavailability (44). On the contrary, the *MKRN3* gene, located within the Prader-Willi syndrome (PWS) region (15q11.2), is the first gene in which loss-of-function mutations have been related to CPP (34). It acts as inhibitor of the pathways leading to puberty beginning, upstream or at the level of kisspeptin and/or GnRH neurons. Low MKRN3 serum levels have been demonstrated before pubertal onset (32, 45) and in girls with CPP compared to controls (35, 46).

Like *MKRN3*, Delta-like 1 homolog (*DLK1*) is a maternally imprinted gene. It is a member of the Notch/Delta/Serrate family belonging to imprinted genes positioned on chromosome 14q32 in humans. This region is associated with Temple syndrome which is characterized by pre- and post-natal growth failure, hypotonia, motor delay and small hands. Interestingly, CPP has been described in 86% of individuals with Temple syndrome (47). *DLK1* is expressed in several tissues during embryonic development, while in postnatal life only in (neuro)endocrine tissues and stem/progenitor cells (48). The Notch signaling pathway is one of the most conserved within species, acting in a context-dependent manner by promoting embryonic cell proliferation and apoptosis (49). Notch signaling is crucial to maintaining the homeostasis in regeneration and damage repair by inducing the differentiation and transformation of mature cells (50). Several ligands and receptors are involved in Notch signaling and have specified temporal and spatial expression in various organs and tissues, including the hypothalamus. How the Notch signaling pathway affects the onset of puberty remains unknown. Recent findings suggest that the Notch regulates progenitor cell differentiation in the pituitary gland, delaying the gonadotrope differentiation (51). The DLK1 intracellular domain has been shown to negatively regulate Notch signaling by disrupting the RBPJ-κ/Notch signaling pathway (52).

Paternally inherited *DLK1* genetic defects have been identified in four families with CPP and metabolic alterations such as obesity, early-onset glucose intolerance, type 2 diabetes mellitus and hyperlipidemia (53, 54). Moreover, *DLK1* mutation has been found to be associated with polycystic ovary syndrome and infertility suggesting a novel link between reproduction and metabolism (54).

The exact role of DLK1 in regulating the timing of puberty is not yet understood; however, DLK1 is likely to regulate hypothalamic neurogenesis and the formation of kisspeptin throughout the activation or inhibition of Notch target genes. Indeed, the Notch signaling pathway could represent a link between the *KISS1*, *MKRN3* and *DLK1* genes.

## Genetic regulation of delayed puberty

Delayed puberty (DP) consists of the absence of pubertal development from the age of 13 years for girls and 14 years for boys. The most frequent phenotype is represented by isolated and self-limiting DP (also described as constitutional retardation of growth and puberty) (55). Most of the subjects with self-limited DP have a family history of late puberty (56, 57). The self-limited DP is inherited in an autosomal dominant, autosomal recessive, or X-linked manner. Furthermore, sporadic cases are also reported. However, few patients with DP have mutations in genes causing abnormalities of the HPG axis, such as *FGFR1*, *GNRHR* and *HS6ST1*, and most of these are relatives of patients with CHH (56–58). Mutations in the Immunoglobulin Superfamily member 10 (*IGSF10)* gene have been found in six unrelated families (59). IGSF10 is important for the appropriate migration of GNRH neurons from the nose to the forebrain during embryonic development. The affected subjects showed pubertal delay without features of constitutional growth delay. A functional defect in the GnRH neuroendocrine system with an increased “threshold” for the onset of puberty, has been hypothesized.

Additionally, loss-of-function mutations in *IGSF10* gene have been found in subjects with hypothalamic amenorrhea (60), suggesting common genetic background with functional central hypogonadism. Subjects affected with both premature ovarian failure and neuronal conditions showed *IGSF10* gene mutations (61). It is not known if these patients also have deficiency of reproductive capacity or sexual lifespan.

To reinforce the concept that the alteration of GnRH neuronal migration during embryonic development can alter pubertal timing, there is a recent preclinical study which demonstrated that the deletion of neuropilin-1 (Nrp1) signaling in GnRH neurons enhances their survival and migration, and their accumulation in the accessory olfactory bulb. In female mice, these alterations result in early prepubertal weight gain, premature attraction to male odors, and precocious puberty (62).

Variants in genes associated with CHH, particularly *GNRHR*, *TAC3* and its receptor *TACR3* have been observed in in cohorts of subjects with self-limited DP (63). However, the pathogenetic role of these variants it is not known. Among other genes involved in the HPG axis function, *LEP* encoding for leptin, *LEPR* encoding for leptin receptor, and *GHSR* encoding for the ghrelin receptor could influence the pubertal timing too. Some studies identified rare variants of these genes; however, it is not clear the association with DP (56).

Pubertal timing seems to be influenced by some genes involved in energy metabolism such as *FTO NEGR1*, *TMEM18* and *SEC16B* genes that have been identified by GWAS (64). Variants of the *FTO* gene have been associated with the regulation of satiety. Rare heterozygous *FTO* variants have been discovered in pedigrees with self-limited DP combined with extreme low BMI by using next generation sequencing (65). Furthermore, knockout mice for the *FTO* gene showed significantly delayed pubertal onset (66).

During the first stages of pubertal development, the loss of the neurobiological brake is managed by several transcription factors organized hierarchically. Therefore, there are transcriptional repressions containing zinc finger motifs that can manage this complex network of genes. The best known are represented by EAP1, Oct-2, Ttf-1, Yy1. EAP1 causes the onset of female puberty through the transactivation of the GnRH promoter. One in-frame deletion (Ala221del) and one rare missense variant (Asn770His) in *EAP1* have been detected in two unrelated families. This condition would result in a reduced transcriptional activity of GnRH resulting in self-limited DP (67).

## Epigenetic control of puberty

The concept of epigenome plasticity explains the adaptation to the environment to regulate the expression of genes that can exert deep effects on the phenotype without modifying the DNA. This reactivity of the epigenome to different signals represents the “epigenetic memory”. Although most of the pathways leading to such changes are still unclear, it is known that these changes can schedule puberty to a specific stage of development (68).

There is evidence on the role of the epigenetic mechanisms in regulating the expression of key actors in the HPG axis, along with its probable role in adapting pubertal timing according to the environment (69).

### DNA methylation

The mechanisms of epigenetic regulation consist in the methylation of CpG (cytosine-guanine) DNA dinucleotides or in the modification of histone proteins (70). DNA methylation and demethylation are catalyzed by DNA methyltransferases (DNMT) and demethylases (TET), respectively, through active or passive mechanisms (71). Active demethylation is an enzymatic reaction that leads to the removal of the 5-methyl group from 5-methyl cytosine through oxidation catalyzed by members of TET family (72). TET2 promotes transcription and peptide release of GnRH thus maintaining reproductive role (73). In addition, DNA methylation and demethylation support the genomic integrity in somatic cells, across silencing or activation of transposable retroelements (REs). The role of DNA methylation in regulating the expression of KNDy system remains unclear (74, 75). DNA methyltransferase inhibitor (DNMTi) has been shown to arrest pubertal onset and this could be reversed by treatment with Kiss1 (76). There are studies showing that the onset of puberty is not regulated by changes in Kiss1 DNA methylation (77, 78). Conversely, although it is not yet clear whether *MKRN3* DNA methylation regulates pubertal onset, some studies proposed a potential role for demethylation-mediated expression of Zinc finger protein 57 (ZFP57) which regulates genomic imprinting (79). The promoter region of the ZFP57 gene is hypomethylated in pubertal girls, and its expression increases in the hypothalamus of female rhesus monkeys at the time of pubertal inception, in line with the increase in KISS1 and GNRH levels (39). Further insights into the epigenetic role of MKRN3 have recently been proven in the MKRN3 knock out mouse which displays CPP. *MKRN3* gene regulates the switch in the onset of mammalian puberty through the ubiquitination of the MBD3 which silences *GNRH1* through disrupting the MBD3 binding to the GNRH1 promoter and recruitment of TET2 (80). These observations support the role of TET2 in direct regulation of GNRH1. Another important regulator of puberty is GnRH receptor (GNRHR) which mediates the GnRH response. The *GNRHR* gene expression is regulated by DNA methylation during neuronal development (81).

Previous studies demonstrated that that Fibroblast growth factor 8 (FGF8) signaling is required for GnRH neuron ontogenesis in the olfactory placode (OP) (82, 83). FGF8 and FGFR1 deficiency is associated with Kallmann Syndrome (KS), a congenital disease characterized by hypogonadotropic hypogonadism and anosmia. Recently, it has been demonstrated that TET1, which converts 5-methylcytosine residues (5mC) to 5-hydroxymethylated cytosines (5hmC), controls transcription of *Fgf8* during GnRH neuron ontogenesis (84). This study demonstrated the importance of epigenetic-dependent timing of *Fgf8* expression during GnRH neuron emergence, and that epigenetic dysfunction can start from the ontogenesis of GnRH neurons onwards and is not limited only to postnatal GnRH neuron organization, potentially contributing to the development of CPP or DP.

### MicroRNAs

GWAS demonstrated an association between menarche age occurrence and *LIN28B* gene polymorphisms, providing the first evidence of an association between miRNAs, epigenetic regulators of gene expression, and pubertal onset (36, 85). Lin28B, and its related Lin28A, are RNA-binding proteins which inhibit the processing of miRNAs of the let-7 family. The role of Lin28 has been confirmed by functional genomic, as transgenic mice overexpressing this protein had overt DP (86). However, the exact repressive mechanism of Lin28 proteins on pubertal development is not known. Furthermore, it is not clear the eventual role of let-7 miRNAs in the central control of puberty.

It has been demonstrated that a microRNA switch regulates the increase in hypothalamic GnRH production before puberty, thus if this event does not occur accurately it may lead to the loss of GnRH expression or alteration of the rhythm of GnRH release and cause hypogonadotropic hypogonadism and infertility in mice (27). Two critical factors of this switch, miR-200 and miR-155, regulate GNRH1 expression through post-transcriptional control of ZEB1 and CEBPB expression, which in turn exert a role in GNRH1 transcriptional repressors in GnRH neurons (27). Recently, in a model of Down syndrome (Ts65Dn mice) the GnRH control appears to be related to an imbalance in a microRNA-gene network which regulate GnRH neuron maturation and hippocampal synaptic transmission (87). Considering the previously mentioned studies on *NOS1* gene alterations, in which both mice and humans show comorbidities such as sensory and cognition impairments, which can be corrected in mice at minipuberty, it can be hypothesized that the maturation of the GnRH system may also play a role in brain development in general, as well in the development of the HPG system.

In addition, miR-7a2 controls the development of the murine pituitary and the function of the HPG axis in mice; thus, its deletion leads to hypogonadotropic hypogonadism and infertility (88). Recently, the expression of miR-411-3p, miR-382-5p, and miR127-3p has been demonstrated to contribute to variability in age at menarche (89).

## Endocrine disruptors and pubertal timing

Endocrine disrupting chemicals (EDCs) are considered responsible of changes in pubertal time (90). Several elements have been recognized as possible EDCs, such as polybrominated biphenyls, bisphenol A (BPA), atrazine (herbicides) (90–92). EDCs can interfere with reproductive functions by mimic or block endogenous hormone function, or by competing with endogenous hormones to bind to carrier proteins (93). Furthermore, they act through G protein-coupled receptors (GPRs) by altering gene expression as well as intracellular signal transduction (94, 95). A relation between early exposure to EDCs and alteration in pubertal timing or concentrations of circulating reproductive hormones has been observed (96–100). They can act in various time windows of development. During fetal life, EDCs can cross the placenta *via* passive or active transport (101–103). The exposure of zebrafish embryos to 17a-ethinylestradiol (EE2) or nonylphenol (NP) disturbs the ontogenesis of GnRH neurons in the forebrain *via* estrogen-receptor pathway (104). In rodents, GnRH neurons use a prostaglandin D2 receptor signaling mechanism during infancy to recruit newborn astrocytes which guide them into adulthood. It has been demonstrated that the exposure to bisphenol A damages postnatal hypothalamic gliogenesis and disrupts the GnRH neurons, impairing minipuberty and delaying the acquisition of reproductive capacity (105). Moreover, epigenetic alterations in testis and other systemic consequences have been observed in pregnant rodents after EDC exposure (106).

Another critical window for EDC exposure is the period of puberty. Kisspeptin neurons are particularly sensitive to early EDC exposure, as mice exposed to low doses of BPA show a reduction in these neurons and a reduced expression of KISS1 and TAC2 in ARC (107). In addition, variations in the pubertal progression have been observed in rodents exposed to EDC during puberty (108, 109).

The exposure to dibutyl phthalate in female rats affects hypothalamic kisspeptin/GPR54 expression determining early puberty and higher levels of serum estradiol (109). Furthermore, EDCs may indirectly damage the transcriptional control of gene expression (110). In pubertal boys and girls, high levels of phthalates in urine have been related with epigenome modifications such as higher DNA methylation levels in the promoter region of the thyroid hormone receptor interactor 6 (*TRIP6*) gene, which regulates pubertal onset (111). Furthermore, children who were exposed to the estrogenic insecticide DTT and then adopted showed precocious puberty (112).

These findings would explain the transgenerational EDC effects. However, although EDCs are known to affect the organization of DNA, the mechanisms by which the epigenetic modifications induced by environmental disruptors are transmitted to the hypothalamic neurons that regulate pubertal initiation must be decoded.

## Conclusions and future perspectives

The genetic and epigenetic mechanisms underlying the physiological variation of the timing of pubertal development are complex and still only partially understood. It may be that some genes act as promoters of the pubertal process, while others act as a brake. Furthermore, early and delayed puberty share some pathogenetic mechanisms, and the epigenetic regulation of the expression of genes involved in pubertal development can begin in fetal life, or during postnatal development and in infancy, with consequent modulation of pubertal time. Reproductive function in humans adapts to adjusting environmental conditions. There are “windows of susceptibility” during the different stages of development that are particularly vulnerable to events or exposures that can determine a long-term reprogramming of the reproductive function of the adult. Furthermore, animal data demonstrate a remarkable sensitivity of the GnRH network to EDCs with the possibility of transmitting phenotypic traits across generations. Molecular and human tissue, animal and cellular models are needed to understand how epigenetic modifications lead to phenotypic variations.

Although recent findings have clarified the influence of epigenetics and mRNAs in the regulation of the pubertal onset, further efforts are needed to better understand how these mechanisms work and which is the role of metabolic and environmental influences, in particular nutritional, on the epigenome. The identification of new neuroendocrine system regulators and the development of preclinical models, together with the application of new technologies for a strict functional activation or inhibition of selected neuronal populations, will be crucial for the acquisition of a deeper mechanistic knowledge of the central systems responsible for the onset of puberty.

## Author contributions

MF did substantial contributions to the conception and design of the work and revised it critically. FU and LAM wrote the first draft of the manuscript. MC and SD wrote sections of the manuscript and prepared the figures; PG revised critically the manuscript. All authors contributed to the article and approved the submitted version.
